# Religion and HPV vaccine-related awareness, knowledge, and receipt among insured women aged 18-26 in Utah

**DOI:** 10.1371/journal.pone.0183725

**Published:** 2017-08-25

**Authors:** Julia Bodson, Andrew Wilson, Echo L. Warner, Deanna Kepka

**Affiliations:** 1 Cancer Control and Population Sciences, Huntsman Cancer Institute, Salt Lake City, Utah, United States of America; 2 Department of Family and Preventive Medicine, University of Utah, Salt Lake City, Utah, United States of America; 3 College of Nursing, University of Utah, Salt Lake City, Utah, United States of America; Rudjer Boskovic Institute, CROATIA

## Abstract

**Introduction:**

We investigate the associations between religious practice and human papillomavirus (HPV) vaccine-related awareness, knowledge, and receipt among young women in Utah.

**Methods:**

We surveyed 326 insured women aged 18–26 by mail. Fisher's Exact Tests and multivariable logistic regression models were used to evaluate the relations between religious practice and HPV vaccine-related outcomes. Data collection occurred January-December 2013; analyses were conducted June-September 2015.

**Results:**

Multivariable analyses reveal that when controlling for age, educational attainment, and marital status, participants who practiced an organized religion were significantly less likely to have heard of HPV (aOR = 0.25, p = 0.0123), to have heard of the HPV vaccine (aOR = 0.41, p = 0.0368), to know how HPV is spread (aOR = 0.45, p = 0.0074), to have received a provider recommendation for the HPV vaccine (aOR = 0.36, p = 0.0332), and to have received at least one (aOR = 0.50, p = 0.0073) or all three (aOR = 0.47, p = 0.0026) doses of the HPV vaccine. Bivariate analyses produce parallel results.

**Conclusions:**

Results indicate that religious young women in Utah are not only under-vaccinated, but are also under-informed about HPV and the HPV vaccine. These results suggest that suboptimal vaccine coverage among religious young women may present a serious health risk for the community. Strategies for educational interventions targeted to this population are discussed.

## Introduction

The Centers for Disease Control and Prevention (CDC) recommends a 3-dose human papillomavirus (HPV) vaccine as a routine vaccination for girls aged 11–12 and as a catch-up vaccination for adolescents and young women aged 13–26 [1]. Though this recommendation has stood for ten years, HPV vaccine coverage in the US remains well below target rates. Only 41.9% of girls aged 13–17 have completed the vaccination series [2], and just 34.5% of young women aged 19–26 have received one dose [3]. With four years to go, the realization of the CDC’s 2020 goal of 80% HPV vaccine coverage seems unlikely [4]. Urgent action is required to address this public health concern [5].

Efforts to improve HPV vaccine uptake have largely focused on adolescents and their parents; however, young women may face a more immediate risk of HPV infection than adolescents, and thus merit serious attention, as well. National survey data indicate that 74% of women are sexually active before their 20^th^ birthday, with 20% of women having their first sexual experience between the ages of 18 and 20 [6]. Moreover, HPV prevalence is highest among young adults, with more than half of 20–24 year-olds infected with at least one strain of HPV [7]. Therefore, HPV vaccination among young women may be a last-chance opportunity for HPV (and HPV-associated cancer) prevention.

It is clearly vital to study the barriers and facilitators to HPV vaccination for young women. Although the research concerning adolescents and their parents is informative, there are substantial differences between parents making vaccination decisions for their children and young adults making vaccination decisions for themselves. Perhaps most importantly, young adults are not uncertain about their own sexual activity in the way that parents may be about their teens’. Indeed, in the few studies examining the HPV vaccination of young women, sexual history was found to be significantly related to vaccination status, intention to vaccinate, and the belief that the vaccine is important [8, 9].

Among the relevant factors that may inform young women’s thinking about the HPV vaccine is their religious beliefs. The connection between HPV and sexual activity has been a major source of religious controversy surrounding the HPV vaccine. Various religious groups have voiced concern that the HPV vaccine will promote sexual disinhibition among teens [10, 11]. Additionally, it has been argued that religious norms regulating sexual activity of unmarried women render the HPV vaccine unnecessary [10–12]. Whereas research examining the association between religion and HPV vaccination among samples of parents has produced mixed results [13–19], the few studies that considered religion in samples of young women have indicated that this factor may be significant [20–23]. Altogether, these findings underscore the importance of exploring the role of religious practice in the HPV vaccination decisions of young women.

Such investigation may be even more salient in religious communities where young women receive information and recommendations from religious health care providers and where community discourse implicates religion directly and indirectly. Utah is a good example of such a community. In Utah, nearly 80% of residents practice an organized religion, with a high density of individuals who identify as members of The Church of Jesus Christ of Latter Day Saints [24]. Furthermore, the boundaries between Church and State in Utah are somewhat porous, and it is speculated that the LDS Church plays a role in shaping state policies, either through legislators’ personal religious views [25], or more directly, as when church leaders weigh in on issues officially [26].

In the present study, we assessed the relations between religion and HPV vaccine-related awareness, knowledge, and receipt among young women in Utah. To the best of our knowledge, there has been no study to date in this community that addresses, in-depth, the association of religion and these HPV vaccine-related outcomes. Our study represents an essential conversation-starter for Utah patients, providers, and policy-makers, and our findings may also be informative for demographically similar communities.

## Methods

This study is part of a larger project for which we surveyed insured women aged 18–26 from University of Utah clinics by mail about their attitudes regarding health and vaccines, as well as about their HPV vaccine-related awareness, knowledge, beliefs, and receipt. Preliminary findings revealed a significant association between religious practice and HPV vaccine receipt [27]; the present study builds on this finding to articulate a more nuanced understanding of the role of religion in HPV vaccination decisions. Data collection took place January 2013-December 2013, and analyses were conducted June 2015-September 2015. The University of Utah Institutional Review Board approved all research.

### Study sample

We recruited participants through the Utah Primary Care Research Network. We identified young women aged 18–26 who had visited a University of Utah Community Clinic in the preceding 12 months (i.e., in the calendar year 2012). Two groups of 1,000 were created; both comprised an outcome-dependent enriched sample of women who had at least one documented dose of the HPV vaccine (Group 1: n = 336; Group 2: n = 233), and a random sample of women with no documented doses of the HPV vaccine (Group 1: n = 664; Group 2: n = 767).

Data collection was conducted in two waves. In the first wave, introduction letters with opt-out information were mailed, followed by the study surveys. The response rate in this wave was below our targets, so we revised methodology using the Tailored Design Method for the data collection among the second group [28], as described previously [27]. In the first wave, we received 85/991 surveys (8.5%). In the second wave, we received 242/901 surveys (27.3%). Combining the two waves, we received a total of 330/1,892 surveys (17.4%). (Only 1,892 surveys were expected after 9 individuals opted-out and 99 surveys were returned undeliverable). Four participants were excluded: two for reporting an age out of range and two for incompleteness of the survey. The remaining 326 participants were included in our analyses. Participants were given full information regarding how completed surveys would be used; consent was implied through returning the surveys. Data can be access in an online data repository (https://osf.io/ajtcw/).

### Measures

The survey (S1 File) evaluated awareness and knowledge related to the HPV vaccine, as well as HPV vaccine receipt. Additionally, the survey assessed attitudes about general health, vaccines, and reproductive health, as well as numerous sociodemographic characteristics. Survey development was informed by the Health Belief Model (HBM) and the Social Cognitive Model (SCM) as described previously [27]. The methods and results of survey validation by Merck were not shared or published. In our previous study using this survey, we found support for a validated instrument in strong factor loadings and high Cronbach’s alpha values within factors [27].

For this study, the predictor of interest was religion, assessed by two questions: “Do you practice an organized religion?” and “Does religion guide your daily decisions?”. The outcomes of interest included HPV vaccine-related awareness (i.e., having heard of HPV and of the HPV vaccine), knowledge (i.e., knowing how HPV is spread and of the relationship between HPV and cervical cancer), and receipt (i.e., receipt of provider recommendation for HPV vaccine, receipt of at least one or all three doses of the HPV vaccine).

### Statistical analysis

Fisher’s Exact Tests and multivariable logistic regression models were used to determine differences in demographics and vaccination rates between participants from the two waves. No statistically significant differences were found, so the participants from both waves were pooled for analyses.

We used Fisher’s Exact Tests to compare distributions between participants who reported practicing vs. not practicing an organized religion on sociodemographic characteristics and on HPV vaccine-related awareness, knowledge, and receipt. We used multivariable logistic regression models to assess the association of religion with HPV vaccine-related outcomes while controlling for potentially confounding variables (age, educational attainment, and marital status). For these tests, we calculated adjusted odds ratios (aOR) and 95% confidence intervals (CIs). We repeated these analyses (bivariate and multivariable) among participants reporting religious practice to compare those who reported that religion does vs. does not guide their daily decisions. For all tests, statistical significance was defined as p<0.05. Analyses were performed using SAS software, version 9.4.

## Results

Participants were fairly evenly distributed by age, were mostly White (n = 263, 80.4%), had mostly graduated or completed some college (n = 252, 77.1%), and were mostly unmarried (n = 232, 71.0%) (Table 1). There were slightly more participants reporting not practicing (n = 178, 54.4%) vs. practicing (n = 148, 45.3%) an organized religion (Table 1). The only significant demographic difference between participants reporting religious practice vs. not was marital status (p<0.0001) (Table 1).

**Table 1:** Demographic characteristics of participants by religious practice (N = 326).

	Practice organized religion (n = 148)	Do not practice organized religion (n = 178)	
	n^a^ (%)^b^	n^a^ (%)^b^	p-value^c^
**Age group**			0.2065
18–21 years old	42 (28.4)	67 (37.6)	
22–24 years old	57 (38.5)	62 (34.8)	
24–26 years old	49 (33.1)	49 (27.5)	
**Race/ethnicity**			0.2177
White	112 (75.7)	151 (84.8)	
Hispanic/Latina	16 (18.8)	9 (5.1)	
African American	3 (2.0)	3 (1.7)	
Asian	10 (6.8)	7 (3.9)	
Other	7 (4.7)	8 (4.9)	
**Educational attainment**			0.5870
≤ High school	19 (12.8)	31 (17.4)	
Some college	58 (39.2)	70 (39.3)	
College graduate	61 (41.2)	63 (35.4)	
Some graduate school	10 (6.8)	14 (7.9)	
**Marital status**			**<0.0001**
Single, never married	84 (57.1)	148 (83.6)	
Ever married	63 (42.9)	29 (16.4)	

^a^ Discrepancies between N = 326 and column totals are the result of missing values in survey responses.

^b^ Percentages calculated out of column totals.

^c^ Boldface indicates statistical significance (p<0.05).

Religious practice was significantly associated with HPV vaccine-related awareness and knowledge (Table 2), specifically with having heard of HPV (p = 0.0092) and of the HPV vaccine (p = 0.0198), and knowing how HPV is spread (p = 0.0062). For each of these outcomes, a higher proportion of participants reporting religious practice reported lack of awareness or knowledge. Religious practice was also significantly negatively related to HPV vaccine receipt (Table 2), specifically to receiving a provider recommendation for the HPV vaccine (p = 0.0196), and to receiving at least one (p<0.0001) or all three (p<0.0001) doses of the HPV vaccine. For these outcomes, too, a higher proportion of participants reporting religious practice reported a lack of recommendation or vaccination.

**Table 2:** HPV vaccine-related awareness, knowledge, beliefs, and receipt among participants by religious practice (N = 326).

	Practice organized religion (n = 148)	Do not practice organized religion (n = 178)	
	n^a^ (%)^b^	n^a^ (%)^b^	p-value^c^
**Heard of HPV**			**0.0092**
Yes	133 (89.9)	173 (97.2)	
No	15 (10.1)	5 (2.8)	
**Heard of the HPV vaccine**			**0.0198**
Yes	127 (86.4)	167 (94.4)	
No	20 (13.6)	10 (5.6)	
**Know how HPV is spread**			**0.0062**
Yes	107 (72.3)	151 (84.8)	
No	41 (27.7)	27 (15.1)	
**Heard of the relationship between HPV and cervical cancer**			0.0531
Yes	110 (74.8)	149 (83.7)	
No	37 (25.2)	29 (16.3)	
**Provider recommended HPV vaccine**			**0.0196**
Yes	80 (85.1)	142 (94.7)	
No	14 (14.9)	8 (5.3)	
**Initiated HPV vaccination series**^d^			**<0.0001**
Yes	76 (48.7)	129 (72.5)	
No	72 (51.4)	49 (27.5)	
**Completed HPV vaccination series**^e^			**<0.0001**
Yes	53 (35.8)	105 (59.0)	
No	95 (64.2)	73 (41.0)	
**Among initiators, intention to complete HPV vaccination series**			0.3368
Yes	14 (60.9)	17 (77.3)	
No	9 (39.1)	5 (22.7)	

^a^ Discrepancies between N = 326 and column totals are the result of missing values in survey responses.

^b^ Percentages calculated out of column totals.

^c^ Boldface indicates statistical significance (p<0.05).

^d^ Initiation defined as reporting receipt of at least 1 dose of HPV vaccination series.

^e^ Completion defined as reporting receipt of 3 doses of HPV vaccination series.

Notably, HPV vaccine-related awareness and knowledge was quite high among all participants, including those reporting religious practice (Table 2). Nearly all participants had heard of HPV (n = 306, 93.6%) and of the HPV vaccine (n = 294, 89.9%); most participants knew how HPV was spread (n = 258, 78.9%) and had heard of the relationship between HPV and cervical cancer (n = 259, 79.2%). Many participants had received a provider recommendation for the HPV vaccine (n = 222, 67.9%), and more than half of participants had received at least one dose of the HPV vaccine (n = 205, 62.7%), including nearly half of the participants reporting religious practice (n = 76, 48.7%) (Table 2). Though fewer participants had completed the vaccination series (n = 158, 48.3%), the rates for participants both reporting religious practice (n = 53, 35.8%) and not (n = 105, 59.0%) were high for this age group (Table 3).

**Table 3:** Multivariable logistic regression for HPV vaccine-related awareness, knowledge, and beliefs.

	Heard of HPV (n = 302)		Heard of the HPV vaccine (n = 290)		Know how HPV is spread (n = 259)	
	aOR^a^ (95% CI)	p-value^b^	aOR^a^ (95% CI)	p-value^b^	aOR^a^ (95% CI)	p-value^b^
**Age**	1.03 (0.81, 1.31)	0.7978	0.95 (0.78, 1.16)	0.6203	0.97 (0.84, 1.12)	0.7037
**Marital status**						
Single, never married	Reference		Reference		Reference	
Ever married	0.73 (0.25, 2.12)	0.5637	0.48 (0.20, 1.17)	0.1047	0.83 (0.43, 1.61)	0.5782
**Educational attainment**						
≤ High school	Reference		Reference		Reference	
Some college	3.22 (0.96, 10.82)	0.0587	3.01 (1.15, 7.91)	**0.0251**	2.25 (1.08, 4.70)	**0.0305**
College graduate	2.69 (0.72, 10.05)	0.1412	5.57 (1.80, 17.27)	**0.0029**	4.23 (1.77, 10.08)	**0.0011**
Some graduate school	3.34 (0.33, 33.63)	0.3058	8.35 (0.92, 76.15)	0.0598	3.330 (0.90, 12.38)	0.0726
**Practice organized religion**						
No	Reference		Reference		Reference	
Yes	0.25 (0.09, 0.74)	**0.0123**	0.41 (0.18, 0.94)	**0.0368**	0.45 (0.25, 0.81)	**0.0074**

^a^ We used simultaneous adjustment for the adjusted odds ratios; that is, the full models calculated for each of the outcomes all include age, marital status, educational attainment, and religious practice as predictors.

^b^ Boldface indicates statistical significance (p<0.05).

Multivariable analyses revealed that when controlling for age, educational attainment, and marital status, religious practice was still significantly associated with certain HPV vaccine-related awareness and knowledge outcomes (Table 3). Compared to participants who reported no religious practice, those who reported religious practice were 4 times less likely to have heard of HPV (aOR = 0.25, 95% CI = 0.09–0.74, p = 0.0123), 2.5 times less likely to have heard of the HPV vaccine (aOR = 0.41, 95% CI = 0.18–0.94, p = 0.0368), and more than 2 times less likely to know how HPV is spread (aOR = 0.45, 95% CI = 0.25–0.81, p = 0.0074). Other variables in these models were also found to be significant (Table 3).

Multivariable analyses showed that religious practice was significantly related to HPV vaccine receipt, as well (Table 4). Compared to their non-religious counterparts, participants who reported religious practice were more than 2.75 times less likely to have received a provider recommendation for the HPV vaccine (aOR = 0.36, 95% CI = 0.14–0.92, p = 0.0332), 2 times less likely to have received at least one dose of the HPV vaccine (aOR = 0.50, 95% CI = 0.30–0.83, p = 0.0073), and more than 2 times less likely to have received all three doses of the HPV vaccine (aOR = 0.47, 95% CI = 0.29–0.78, p = 0.0026). Again, other variables in the models were also found to be significant (Table 4).

**Table 4:** Multivariable logistic regression for HPV vaccine receipt.

	Provider recommended HPV vaccine (n = 223)		Initiated HPV vaccine^a^ (n = 206)		Completed HPV vaccine^b^ (n = 159)	
	aOR^c^ (95% CI)	p-value^d^	aOR^c^ (95% CI)	p-value^d^	aOR^c^ (95% CI)	p-value^d^
**Age**	0.79 (0.61, 1.01)	0.0599	0.90 (0.79, 1.02)	0.1012	0.88 (0.78, 1.00)	0.0528
**Marital status**						
Single, never married	Reference		Reference		Reference	
Ever married	1.02 (0.35, 2.93)	0.9748	0.38 (0.21, 0.67)	**0.0008**	0.43 (0.24, 0.77)	**0.0048**
**Educational attainment**						
≤ High school	Reference		Reference		Reference	
Some college	1.78 (0.41, 7.67)	0.4411	2.27 (1.12, 4.68)	**0.0257**	2.70 (1.31, 5.59)	**0.0074**
College graduate	1.82 (0.39, 8.53)	0.4486	2.62 (1.18, 5.82)	**0.0178**	3.19 (1.40, 7.25)	**0.0057**
Some graduate school	3.36 (0.28, 40.70)	0.3413	1.27 (0.42, 3.90)	0.6727	2.08 (0.66, 6.63)	0.2136
**Practice organized religion**						
No	Reference		Reference		Reference	
Yes	0.36 (0.14, 0.92)	**0.0332**	0.50 (0.30, 0.83)	**0.0073**	0.47 (0.29, 0.78)	**0.0026**

^a^ Initiation defined as reporting receipt of at least 1 dose of HPV vaccination series.

^b^ Completion defined as reporting receipt of 3 doses of HPV vaccination series.

^c^ We used simultaneous adjustment for the adjusted odds ratios; that is, the full models calculated for each of the outcomes all include age, marital status, educational attainment, and religious practice as predictors.

^d^ Boldface indicates statistical significance (p<0.05).

Of the 148 respondents who reported practicing an organized religion, 103 (69.6%) responded that religion guides their daily decisions, and 41 (27.7%) said it did not (n = 4 missing response). Analyses to compare religious participants who reported that religion does vs. does not guide their daily decisions yielded few significant results (data not shown). The sole demographic difference between these participants was educational attainment (p = 0.0039). We found no significant relation in either bivariate or multivariable analyses for any HPV vaccine-related outcome.

## Discussion

Though the HPV vaccine has been recommended for girls and young women for a decade [1], coverage remains low in the US [2, 3]. Uptake has been particularly poor in Utah [2], a state with a dense [24] and potentially influential [25, 26] religious population. The religious climate in Utah may underlie low vaccination rates, and could also affect HPV vaccine-related awareness and knowledge. Our study is the first to investigate the relations between religion and these HPV vaccine-related outcomes among young women in Utah. Though participants had high HPV vaccine-related awareness, knowledge, and receipt overall, our findings reveal considerable differences between religious and secular young women. These results may be used in Utah, as well as in other densely religious populations, to inform interventions geared toward improving HPV vaccine-related awareness and knowledge, as well as HPV vaccination.

Our findings indicate that participants who practiced an organized religion were significantly less likely to have received a recommendation for, and to be vaccinated with, the HPV vaccine, even when controlling for age, education, and marital status. These findings reinforce results of previous studies that demonstrated significant associations between religion and the HPV vaccination and vaccination intention of young women [21–23]. However, it should be noted that these differential vaccination outcomes in and of themselves need not cause immediate concern. It is plausible that religious participants are merely at a lower risk for being infected with HPV due to limited risky sexual activity [29]. That is, if religious participants had discussed the costs and benefits of the vaccine with their doctors, and based their decision on their doctors’ recommendations, these findings would not reflect a significant health risk.

However, our findings also reveal that religious participants were significantly less likely than their non-religious counterparts to be aware of or knowledgeable about the HPV vaccine. Religious participants were less likely to have heard of HPV and the HPV vaccine, and were also less likely to know how HPV is transmitted. These findings are novel in regards to this population; only one previous study has suggested that religious affiliation significantly relates to awareness and knowledge of HPV [20]. In light of these findings, the differential vaccination outcomes can no longer be thought of as unproblematic. On the contrary, these findings suggest that the decision of religious young women to not vaccinate may not be based on a complete understanding of the risk or the vaccine. Put differently, the religious participants in this study were less likely to exhibit a basic awareness of the virus, the vaccine, or the mechanism of transmission, revealing that their decisions not to vaccinate may not necessarily have been informed. Our findings may expose a failure of providers to inform their patients, as well as a failure of patients to educate themselves.

Finally, unlike the more straightforward question regarding whether participants practice an organized religion, the more subjective question about whether religion guides daily decisions yielded no significant findings. This may be because religious individuals are unaware of the extent to which religion informs their daily decisions, or because they do not think of decisions about HPV vaccination as “daily.” But perhaps the workings of religion do not occur via the subjective sense of being guided by it. Perhaps religious individuals, regardless of whether or not they feel that their religion guides their daily decisions, tend to choose doctors who endorse the same religious beliefs, participate in social networks with people of the same religion, and access media created by and for people of the same religion. Thus, whether or not they acknowledge that religion actively guides their daily decisions, the mere and actual practice of an organized religion may structure their lives in ways that significantly affect their HPV vaccine-related decisions and HPV vaccine-related outcomes.

In sum, our findings document the significantly lower HPV vaccine-related awareness, knowledge, and receipt among young women who practice an organized religion compared to those who do not. The results signal the need for interventions geared to, at the very least, educating religious young women in Utah about HPV and the HPV vaccine. Such educational interventions are critical to ensure that a decision to not vaccinate is based on full and accurate information, thereby representing a low health risk. To create and enact such interventions will require the participation of a variety of stakeholders, including religious young women, their health care providers, church leaders, policy makers, and public health officials. Notably, our findings also suggest that it will be important to reach out broadly to religious young women, including those who may not feel as though religion guides their daily decisions. This could be accomplished by tailoring materials for religious audiences both explicitly (e.g., using religious quotations) and implicitly (e.g., using motivational messages), and by targeting religious individuals directly (e.g., in church-based groups) or indirectly (e.g., via media campaigns hosted on networks popular among religious populations). Interventions focused on medical providers who serve religious patients may also be effective—for example, through requirements to provide standardized HPV vaccine education.

### Limitations

Participants were mostly white and were all insured, limiting the generalizability of our findings. Given that this study took place in a densely and fairly homogeneous religious population, the results may not generalize to more diverse communities. It is also worth noting that our sample exhibited lower rates of religious practice than the overall Utah population; therefore, our findings may not fully represent the population from which we sampled. The study may also suffer from response bias; we had a limited sample size, and the response rate for the survey was poor, especially in the first wave of data collection. We also received more responses from participants who were vaccinated vs. unvaccinated, suggesting a voluntary response bias. Additionally, the cross-sectional study design prohibits the evaluation of causal relationships. We did not assess specific religious affiliation (only religious practice), so we cannot be certain as to which religious groups our results may apply. Moreover, we did not assess the sexual history of participants, which could have been a significant confounding variable within this analysis.

## Conclusions

This study is, to the best of our knowledge, the first to explore the relations between religion and HPV vaccine-related awareness, knowledge, and receipt among young women in Utah. Not only was HPV vaccination significantly lower among religious individuals, so was HPV vaccine-related awareness and knowledge. These results must inform educational interventions to raise HPV vaccine-related awareness and knowledge among religious young women in Utah, including those for whom religion does not guide daily decisions. These interventions will require the participation of a variety of stakeholders, and may take the form of church-based education groups, targeted media campaigns, or provider-focused efforts.

## Supporting information

S1 File Survey booklet. (PDF)
