# Supplementary material for: Religion and HPV vaccine-related awareness, knowledge, and receipt among insured women aged 18-26 in Utah
Source: PLoS One. 2017 Aug 25;12(8):e0183725. doi: 10.1371/journal.pone.0183725 (PMC5571930; doi:10.1371/journal.pone.0183725)
Supplement: S1 File — (PDF) [file pone.0183725.s001.pdf]

Survey Booklet

18-26 Year Old Women

SURVEY INSTRUCTIONS

This survey will ask you a series of questions about women's health issues, vaccinations, and awareness of cervical cancer and how to help prevent this disease. It should take approximately 15-20 minutes to complete. For each question, please read the instructions and use a pen or pencil to mark the box with an "X" that best corresponds to your answer.

For some questions, you may be asked to skip to another question based on the answer you choose. When this happens you will see an arrow that tells you which question to answer next. Please follow these instructions carefully.

SECTION 1: ATTITUES ABOUT YOUR HEALTH

1. First, we want to ask you about your health. Below is a list of statements which may or may not describe your attitudes about your health. Please read each statement and indicate how much you agree or disagree with it by marking the appropriate box with an "X".

	Strongly Disagree	Moderately Disagree	Slightly Disagree	Slightly Agree	Moderately Agree	Strongly Agree
a. If I get sick, it is my own behavior which determines how soon I get well again.	1 ☐	2 ☐	3 ☐	4 ☐	5 ☐	6 ☐
b. No matter what I do, if I am going to get sick, I will get sick.	1 ☐	2 ☐	3 ☐	4 ☐	5 ☐	6 ☐
c. Having regular contact with my physician is the best way for me to avoid illness.	1 ☐	2 ☐	3 ☐	4 ☐	5 ☐	6 ☐
d. I trust the medical community has my best interests in mind.	1 ☐	2 ☐	3 ☐	4 ☐	5 ☐	6 ☐
e. Whenever I don't feel well, I should consult a medically trained professional.	1 ☐	2 ☐	3 ☐	4 ☐	5 ☐	6 ☐
f. I am in control of my health.	1 ☐	2 ☐	3 ☐	4 ☐	5 ☐	6 ☐
g. My family has a lot to do with my becoming sick or staying healthy.	1 ☐	2 ☐	3 ☐	4 ☐	5 ☐	6 ☐
h. When I get sick I am to blame.	1 ☐	2 ☐	3 ☐	4 ☐	5 ☐	6 ☐
i. Luck plays a big part in determining how soon I will recover from an illness.	1 ☐	2 ☐	3 ☐	4 ☐	5 ☐	6 ☐
j. Health professionals control my health.	1 ☐	2 ☐	3 ☐	4 ☐	5 ☐	6 ☐

		Strongly Disagree	Moderately Disagree	Slightly Disagree	Slightly Agree	Moderately Agree	Strongly Agree
k.	My good health is largely a matter of good fortune.	1☐	2☐	3☐	4☐	5☐	6☐
l.	The main thing which affects my health is what I myself do.	1☐	2☐	3☐	4☐	5☐	6☐
m.	If I take care of myself, I can avoid illness.	1☐	2☐	3☐	4☐	5☐	6☐
n.	No matter what I do I'm likely to get sick.	1☐	2☐	3☐	4☐	5☐	6☐
o.	If it's meant to be, I will stay healthy.	1☐	2☐	3☐	4☐	5☐	6☐
p.	If I take the right actions, I can stay healthy.	1☐	2☐	3☐	4☐	5☐	6☐
q.	Regarding my health, I can only do what my doctor tells me to do.	1☐	2☐	3☐	4☐	5☐	6☐

SECTION 2: ATTITUES ABOUT VACCINES

2. These next questions ask about your opinions regarding vaccines (shots to help prevent disease). Please read each statement and indicate how much you agree or disagree with it by checking the appropriate box.

		Strongly Disagree	Moderately Disagree	Slightly Disagree	Slightly Agree	Moderately Agree	Strongly Agree
a.	Vaccines are a good way to protect public health.	1☐	2☐	3☐	4☐	5☐	6☐
b.	I do not like the idea of vaccines.	1☐	2☐	3☐	4☐	5☐	6☐
c.	Shots are very painful.	1☐	2☐	3☐	4☐	5☐	6☐
d.	Vaccines are generally safe.	1☐	2☐	3☐	4☐	5☐	6☐
e.	Getting vaccinated can make you sick.	1☐	2☐	3☐	4☐	5☐	6☐
f.	I am not afraid of shots.	1☐	2☐	3☐	4☐	5☐	6☐
g.	Vaccines are a way to take good care of myself now and in the future.	1☐	2☐	3☐	4☐	5☐	6☐
h.	Vaccines are effective.	1☐	2☐	3☐	4☐	5☐	6☐

SECTION 3: ABOUT YOU

The next questions ask you some general questions about yourself. Your answers will help us describe the types of women participating in this study. Please read each question carefully and respond by marking the appropriate boxes with an "X".

3a. Have you ever received a cancer diagnosis?

1☐ Yes

2☐ No

3b. Do you know anyone who has had a cancer diagnosis?

1☐ Yes ➔ *Go to Question 3c.*
2☐ No ➔ *Go to Question 4.*

3c. Was it cervical cancer?

1☐ Yes
2☐ No

4. In what year were you born? Please enter as a four-digit number (e.g., 1983).

Year of birth: | 1 | 9 | | |

5. Which of the following do you consider yourself to be? *(Check all that apply)*

1☐ American Indian or Alaska Native
2☐ Asian
3☐ Black or African American
4☐ Hispanic or Latino
5☐ Native Hawaiian or Other Pacific Islander
6☐ White
7☐ Other: ______________________________

6. What is the highest level of education you have completed or the highest degree you have received?

1☐ Less than high school
2☐ Some high school
3☐ High school or equivalent (e.g., GED)
4☐ Some college, but no degree
5☐ Two-year degree (community or technical)
6☐ College graduate
7☐ Graduate school

7. What is your marital status?

1☐ Single, never married
2☐ Married
3☐ Divorced
4☐ Separated
5☐ Widowed

8. Do you practice organized religion?

1☐ Yes ➔ *Go to Question 9.*
2☐ No ➔ *Go to Section 4.*

9. Does religion guide your daily decisions?

1☐ Yes
2☐ No

SECTION 4: ATTITUDES ABOUT REPRODUCTIVE HEALTH

This section of the survey consists of a list of statements, which may or may not describe your attitudes about reproductive health including sexual health. Some statements may contain descriptions of medical tests that are unfamiliar to you. Please use these definitions below to help you answer the questions in this section.

- **Pap test/Pap smear:** A procedure in which cells are scraped from the cervix for examination under a microscope. It is used to detect cancer and changes that may lead to cancer. A Pap smear can also show noncancerous conditions, such as infection or inflammation.
- **Gynecological/pelvic examination:** A physical examination in which the health care professional will feel for lumps or changes in the shape of the vagina, cervix, uterus, fallopian tubes, ovaries, and rectum. The health care professional will also use a speculum to open the vagina to look at the cervix and take samples for a Pap test. This may also be called an internal exam.

10. Have you ever heard of human papillomavirus (HPV)?

1☐ Yes
2☐ No

11. Do you know how HPV is spread?

1☐ Yes
2☐ No

12. Please read each statement and indicate how much you agree or disagree with it by marking the appropriate box with an "X". If you are uncertain about how to answer a specific question, please just choose the response that comes closest to your opinion. If a particular question does not apply to you, please write "NA" (not applicable) next to the question.

		Strongly Disagree	Moderately Disagree	Slightly Disagree	Slightly Agree	Moderately Agree	Strongly Agree
a.	I am comfortable discussing sexual health issues with a doctor or nurse.	1☐	2☐	3☐	4☐	5☐	6☐
b.	I am comfortable discussing sexual health issues with others such as family or friends.	1☐	2☐	3☐	4☐	5☐	6☐
c.	A physician's recommendation to receive the HPV vaccine would influence me.	1☐	2☐	3☐	4☐	5☐	6☐
d.	I don't mind getting a gynecological/pelvic exam.	1☐	2☐	3☐	4☐	5☐	6☐

		Strongly Disagree	Moderately Disagree	Slightly Disagree	Slightly Agree	Moderately Agree	Strongly Agree
e.	Gynecological/pelvic exams are necessary to stay healthy.	1☐	2☐	3☐	4☐	5☐	6☐
f.	I get a Pap test/Pap smear according to my doctor's/health care provider's advice.	1☐	2☐	3☐	4☐	5☐	6☐
g.	It is very important to have an annual pelvic exam.	1☐	2☐	3☐	4☐	5☐	6☐
h.	Vaccines are safe. In particular, HPV vaccine is safe.	1☐	2☐	3☐	4☐	5☐	6☐
i.	Cervical cancer is a devastating disease.	1☐	2☐	3☐	4☐	5☐	6☐
j.	Genital warts are an embarrassing condition.	1☐	2☐	3☐	4☐	5☐	6☐

SECTION 5: ATTITUES ABOUT HPV VACCINE

Next you will be asked a series of questions about vaccinations to help protect against cervical cancer and human papillomavirus (HPV) infection. Please read each question carefully and respond by marking the appropriate box with an "X".

13. Have you ever heard of a relationship between HPV and cervical cancer?

1☐ Yes
2☐ No

14. Have you ever heard of a vaccine to prevent HPV (e.g., Gardasil® or Cervarix®)?

1☐ Yes ➔ *Go to Question 15*
2☐ No ➔ **STOP - You have reached the end of the survey.**
Please return this completed booklet.
Thank you for your participation.

15. If you answered "Yes" to Question 14 about having heard of Gardasil® or Cervarix®, how important do you think the vaccine to help prevent cervical cancer is for you?

1☐ Not at all important
2☐ Not very important
3☐ Somewhat important
4☐ Very important

16. Have you discussed the vaccine to help prevent cervical cancer with a doctor?

1☐ Yes ➔ *Go to Question 17*
2☐ No ➔ Skip to SECTION 6: FUTURE CERVICAL CANCER VACCINE USE

17. Did a doctor recommend that you get the vaccine to help prevent cervical cancer?

1☐ Yes ➔ *Go to Question 18*
2☐ No ➔ Skip to SECTION 6: FUTURE CERVICAL CANCER VACCINE USE

18. If you answered "Yes" to Question 17 about a doctor recommending the cervical cancer vaccine, how strongly did the doctor recommend you get the cervical cancer vaccine on a scale from 1 to 5?

1☐ **A doctor did not** strongly recommend the vaccine
2☐
3☐
4☐
5☐ **A doctor strongly** recommended the vaccine

19. Have you received one or more doses of the vaccine to help prevent cervical cancer?

1☐ Yes ➔ *Go to Question 20*
2☐ No ➔ Skip to SECTION 6: FUTURE CERVICAL CANCER VACCINE on the next page.

20. The full course of the vaccine to help prevent cervical cancer requires three doses in total. How many doses of the cervical cancer vaccine have you received to date?

1☐ One dose
2☐ Two doses
3☐ Three doses ➔ **If you answered three doses, STOP – You have reached the end of the survey. Please return this completed booklet. Thank you for your participation.**

21. If you have not received all three doses yet, do you intend to do so?

1☐ Yes ➔ *Skip to Question 23*
2☐ No ➔ *Go to Question 22*

22. Why do you not intend to receive all three doses? *(Check all that apply)*

1☐ Cost of the vaccine
2☐ I had a reaction to the vaccine
3☐ I found the injection to be too painful
4☐ Vaccine was inconvenient to get
5☐ I worry about the vaccine's safety
6☐ I got pregnant
7☐ I got married
8☐ I am no longer at risk of human papillomavirus (HPV) infection
9☐ Other (describe): ____________________

If you answered Question 22, then STOP, you have reached the end of the survey. Please return this completed booklet. Thank you for your participation.

SECTION 6: FUTURE HPV VACCINE USE

Please answer these final questions only if you have never received a dose of cervical cancer vaccine. Read each question carefully and mark the appropriate answer with an "X".

23. If you have not received a single dose of the cervical cancer vaccine, in the future how likely are you to:

	Not at all likely	Not very likely	Somewhat likely	Very likely	Extremely likely
a. Ask a doctor to get this vaccine?	1☐	2☐	3☐	4☐	5☐
b. Do additional research on this vaccine?	1☐	2☐	3☐	4☐	5☐
c. Discuss the vaccine with a doctor?	1☐	2☐	3☐	4☐	5☐
d. Make an appointment to get the vaccine?	1☐	2☐	3☐	4☐	5☐
e. Do nothing to get the vaccine?	1☐	2☐	3☐	4☐	5☐

24. If you answered that you are somewhat likely, very likely or extremely likely to "Do nothing to get the vaccine" in Question 23e above, please indicate the reason(s) why you would not do anything. *(Check all that apply)*

1☐ I am not sexually active
2☐ I am waiting for more information - the vaccines are still too new
3☐ I am married or in a exclusive (monogamous) relationship
4☐ I am unsure if my insurance would cover the vaccine cost
5☐ I do not have enough information about this vaccine
6☐ I am concerned about the side effects
7☐ I am pregnant or trying to conceive
8☐ It is not socially acceptable to receive the vaccine
9☐ My doctor recommended against getting the vaccine
10☐ I cannot afford the cost of the vaccine
11☐ The HPV vaccination would not affect *my* risk of HPV
12☐ My religion prohibits or strongly discourages vaccinations
13☐ My religion prohibits or strongly discourages HPV in particular
14☐ Other (describe): ______________________________

25. Would any of the following influence your decision about the HPV vaccination? *(Check all that apply)*

1☐ Physician
2☐ Spiritual or religious leader
3☐ Friend or coworker
4☐ Family member
5☐ Other (describe): ______________________________

THANK YOU! You have reached the end of the survey.
Please review your responses to make sure all questions have been answered.
